# TgICMAP1 Is a Novel Microtubule Binding Protein in *Toxoplasma gondii*

**DOI:** 10.1371/journal.pone.0007406

**Published:** 2009-10-12

**Authors:** Aoife T. Heaslip, Stephanie C. Ems-McClung, Ke Hu

**Affiliations:** 1 Department of Biology, Indiana University, Bloomington, Indiana, United States of America; 2 Medical Science Program, Indiana University, Bloomington, Indiana, United States of America; University of Minnesota, United States of America

## Abstract

The microtubule cytoskeleton provides essential structural support for all eukaryotic cells and can be assembled into various higher order structures that perform drastically different functions. Understanding how microtubule-containing assemblies are built in a spatially and temporally controlled manner is therefore fundamental to understanding cell physiology. *Toxoplasma gondii*, a protozoan parasite, contains at least five distinct tubulin-containing structures, the spindle pole, centrioles, cortical microtubules, the conoid, and the intra-conoid microtubules. How these five structurally and functionally distinct sets of tubulin containing structures are constructed and maintained in the same cell is an intriguing problem. Previously, we performed a proteomic analysis of the *T. gondii* apical complex, a cytoskeletal complex located at the apical end of the parasite that is composed of the conoid, three ring-like structures, and the two short intra-conoid microtubules. Here we report the characterization of one of the proteins identified in that analysis, TgICMAP1. We show that TgICMAP1 is a novel microtubule binding protein that can directly bind to microtubules *in vitro* and stabilizes microtubules when ectopically expressed in mammalian cells. Interestingly, in *T. gondii*, TgICMAP1 preferentially binds to the intra-conoid microtubules, providing us the first molecular tool to investigate the intra-conoid microtubule assembly process during daughter construction.

## Introduction

The microtubule (MT) cytoskeleton provides structural support for vital functions such as cell motility, cell division, and material flow in eukaryotic cells. A MT is a polymer with distinct polarity, where each MT subunit, i.e. alpha and beta tubulin heterodimer, is placed in the same orientation in the polymer, resulting in two ends (minus and plus ends) that have different structure and assembly dynamics. Higher order structures formed by MT and MT-binding proteins perform drastically different functions such as flagella beating, chromosome alignment and segregation, and vesicular transport. Understanding how MT-containing assemblies are built in a spatially and temporally controlled manner is fundamental to understanding cell physiology.

**Figure 1:**
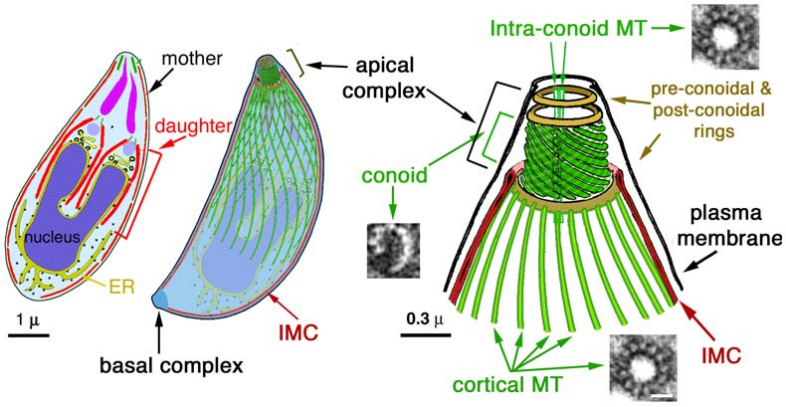
Schematic drawings of *T. gondii* (Adapted from [6]). *Left and middle*: a longitudinal section and a semitransparent projected view of the cytoskeleton of a dividing cell. *Right*: an enlarged view of the apical portion of the cytoskeleton. The cytoskeleton of *T. gondii* includes the apical and the basal complexes, and the cortical cytoskeleton, which contains the cortical MTs, and the Inner Membrane Complex (IMC), a set of flattened vesicles with an underlying filamentous protein network. The centrioles/spindle pole assembly is located close to the nucleus and is not shown in the drawings. The apical complex is formed of the conoid, the intra-conoid MTs and three ring-like structures. The conoid fibers are novel tubulin polymers, the structure of which is dramatically different from that of a canonical MT, such as the cortical MTs and the intra-conoid MTs. In the enlarged view of the apical portion of the cytoskeleton (*right*), EM cross-sections of a single conoid fiber and a canonical MT are shown.

*Toxoplasma gondii* is a fantastic cell biological model organism for studying the biogenesis of tubulin containing structures. It has a strictly reproducible shape, defined by a highly ordered cytoskeleton maintained precisely through generations. The ultrastructure of *T. gondii* cytoskeleton is very well characterized [1]–[7]. The cytoskeleton of every single parasite in a population contains exactly 22 cortical MTs, one basal complex, and one apical complex made of three ring structures, 14 filaments of a novel tubulin polymer (collectively termed “the conoid”) [7], and two intra-conoid MTs. There are at least five distinct tubulin-containing structures within *T. gondii* (Figure 1). Besides the conoid, intra-conoid MTs, and cortical MTs, MTs are also assembled into two other structures: spindle poles, which organize the intra-nucleus spindle during parasite replication, and centrioles, which contain a central single MT and nine outer single MTs [4]. This MT organization of the centrioles is distinct from the centrioles of higher eukaryotes that are composed of two central MTs and nine triplet peripheral MTs. The parasite also contains another novel cytoskeletal structure, termed the basal complex, at its basal end [6], [8]. The basal complex contains a number of putative MT binding proteins such as dynein light chain and centrin 2, but no tubulin based structures have been observed in the basal complex.

How *T. gondii* constructs and maintains the five structurally and functionally distinct sets of tubulin containing structures in the same cell is an intriguing problem. For example, both the intra-conoid MTs and cortical MTs are canonical MTs with 13 protofilaments; however, their localization and length are very different. The 22 cortical MTs originate from a ring like structure at the distal end of the apical complex, and extend for ∼5 µm, or ∼2/3 of the parasite body [1], [3], [4], [7]. The two intra-conoid MTs, however, do not have an obvious organizing center. They are positioned close to the center of the apical complex, and only about 0.4 µm in length. CryoEM studies show that both sets of MTs are heavily decorated with MT binding proteins with quite different association patterns [1], [7]. The identification and characterization of MT binding proteins, therefore, is key to understanding the construction and functions of these various MT-containing assemblies. Finally, any new knowledge concerning *T. gondii* cytoskeleton is particularly welcome, as *T. gondii* is one of the most prevalent parasites in warm-blooded animals and is the most common cause of congenital neurological defects in humans [9]. It also causes devastating opportunistic infections in immuno-compromised patients [10]. Many of its 5,000 relatives in phylum *Apicomplexa* are also important human or animal pathogens [11], including *Plasmodium spp*, which kills more than a million people every year. The damage caused by *T. gondii*, most commonly severe lytic cerebral and ocular lesions, absolutely depends on its ability to replicate [10], [12]–[14]. In the absence of massive, uncontrolled expansion of the parasite population, the infections are benign [15]–[17]. The MT-based *T. gondii* cytoskeleton is essential for parasite survival and proliferation, as it provides the framework for organelle replication and partition. In addition, it is likely to be important for parasite invasion. The intra-conoid MTs, for example, are closely associated with the secretory organelles, such as rhoptries and micronemes, and are speculated to provide structural support for protein secretion from these organelles during invasion [3], [18]. As tubulins themselves are highly conserved between *T. gondii* and mammalian cells [19], novel MT binding proteins in *T. gondii* are prime drug targets.

Here we report the identification and characterization of a novel *T. gondii* MT binding protein, intra-conoid microtubule associated protein1 (TgICMAP1, Accession number: TgME49_039300). TgICMAP1 is a large 135 kDa protein that contains a coiled-coil Structural Maintenance of Chromosomes (SMC)-like domain. Purified recombinant eGFP-TgICMAP1 protein binds to *in vitro* polymerized MTs, suggesting that its interaction with the MT is direct. Surprisingly, in *T. gondii*, TgICMAP1 preferentially binds to the intra-conoid MTs. When over-expressed in mammalian cells, eGFP-TgICMAP1 coats and stabilizes MTs, suggesting that TgICMAP1 might play a role in stabilizing the intra-conoid MTs. The discovery of TgICMAP1 thus provides us the first molecular tool to dissect the function and the assembly pathway of the intra-conoid MTs.

## Results

### TgICMAP1 is a coiled-coil protein that binds to and stabilizes MTs when ectopically expressed in mammalian cells

**Figure 2:**
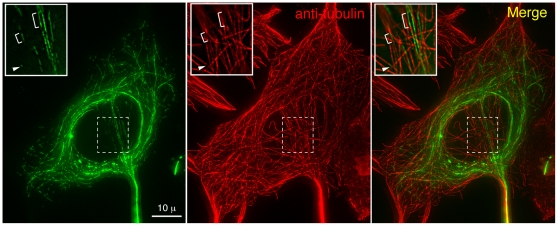
eGFP-TgICMAP1 coats MTs when ectopically expressed in mammalian cells. Images of mouse embryonic fibroblast cells transiently expressing eGFP-TgICMAP1 (*green*) labeled with anti-tubulin YL1/2 (*red*), which show that eGFP-TgICMAP1 binds to a subset of MTs, mainly around the nucleus. *Inset*s: notice that MT segments labeled by eGFP-TgICMAP1 tend to have much weaker staining of tubulin antibody than adjacent segments (arrowheads and brackets). Images are projections of deconvolved 3-D stacks. Insets are single optical sections of indicated regions in images and are at 2× magnification.

Despite the richness of novel tubulin containing structures in *T. gondii*, only a few MT binding proteins have been identified in *T. gondii* [6]. Previously, we identified ∼170 proteins in the apical complex of *T. gondii* using comparative proteomics [6]. To quickly assess if any of the novel proteins could interact with MTs, eGFP fusions of a number of these proteins were constructed and expressed in mammalian cells. One of the proteins tested, designated TgICMAP1, showed binding along the lattice of MTs (Figure 2). TgICMAP1 is likely to have a high affinity for MTs, as heavy eGFP-TgICMAP1 coating correlated with much weaker anti-tubulin staining when compared with that of uncoated MT segments, suggesting that perhaps TgICMAP1 binding blocks the accessibility of tubulin antibody to the MTs (Figure 2, insets). To determine if TgICMAP1 homologues are present in other protozoan parasites species, the TgICMAP1 protein sequence was blasted against the available apicomplexan parasite genomic sequences (http://eupathdb.org/eupathdb/), including those of *Plasmodium spp*, *Cryptosporidium spp*, *Theileria spp*, *and Neospora caninum*. The search revealed a single homologue in one of the closest relatives of *T. gondii*, *Neospora caninum*, another parasite that belongs to the family of *Sarcocystidae*. *Neospora* ICMAP1 (NcICMAP1, Accession Number: Nc_LIV_ 070470) shares 82% identity and 89% similarity with TgICMAP1 (Figure S1).

**Figure 3:**
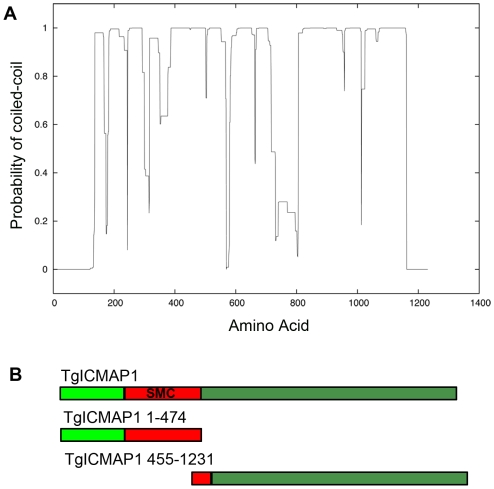
TgICMAP1 is a coiled-coil protein and contains a SMC-like coiled-coil domain. (A) Graphic display of predicted coiled-coil domains in TgICMAP1. Y-axis, probability of formation of coiled-coil domain where values close to 1 indicate regions with a high propensity to form coiled-coil domains. X-axis, TgICMAP1 amino acids. (B) Schematics of full-length TgICMAP1 and the two TgICMAP1 truncation proteins, TgICMAP1^1–474^ and TgICMAP1^455–1231^.

Approximately 50% of TgICMAP1 was predicted to form coiled-coil domains by coiled-coil domain prediction software (Figure 3A) [20]. When blasted against the NCBI non-redundant protein database, the sequence from amino acids 200 to 475 in TgICMAP1 was recognized to be homologous to the coiled-coil region in Structural Maintenance of Chromosomes (SMC) domains (EValue = 4e^-09^) (Figure 3B and S1) [21]–[26]. The ATPase domain in typical SMC proteins is absent in TgICMAP1, but the presence of the coiled-coil region in the SMC domain internal to TgICMAP1 may suggest anti-parallel dimerization of this molecule through intermolecular association [21].

**Figure 4:**
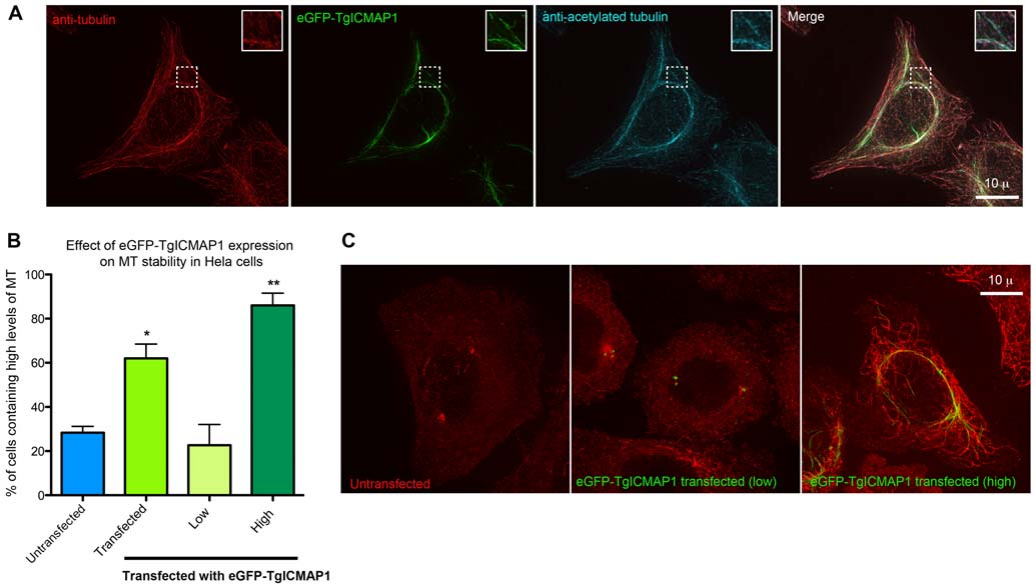
Ectopically expressed eGFP-TgICMAP1 stabilizes MTs in mammalian cells. (A) eGFP-TgICMAP1 transfected cells (*green*) labeled with rat anti-tubulin YL1/2 antibody (*red*) and mouse anti-acetylated tubulin antibody (*cyan*). eGFP-TgICMAP1 coated MTs display higher acetylation level compared with adjacent “uncoated” MTs (*insets*). Insets are at 2× magnification. Scale bar = 10 µm. (B) Graph showing the mean percentages of untransfected (blue bar, “untransfected”); eGFP-TgICMAP1 transfected (green bar, “transfected”); low (yellow bar) and high (dark green bar) eGFP-TgICMAP1 expressing HeLa cells containing high levels of MTs after nocadazole treatment. The error bars indicate standard error of the mean (SEM). “*” : p<0.01, “**”: p<0.001. (C) Representative images of untransfected and transfected cells expressing low and high levels of eGFP-TgICMAP1 after nocodazole treatment. Scale bar = 10 µm.

When expressed at a high level, eGFP-TgICMAP1 colocalizes with perinuclear MTs (Figure 2). To determine if there is any correlation between TgICMAP1 binding and tubulin post-translational modifications, immunofluorescence experiments with anti-acetylated tubulin antibodies were performed. Analysis showed that MTs that were heavily coated with eGFP-TgICMAP1 were also acetylated (Figure 4A). Since acetylation is often a marker for stable MTs [27]–[29], we wanted to directly test if the binding of eGFP-TgICMAP1 indeed increases MT stability. Upon treatment with 2 µM nocodazole for four hours, 62% of HeLa cells transfected with eGFP-TgICMAP1 still contained high levels of MTs, significantly higher than that of untransfected cells on the same slide, which was 28% (p<0.01) (Figure 4, B and C). High eGFP-TgICMAP1 expression level accentuated this effect, as 86% of cells expressing high level of eGFP-TgICMAP1 (defined by fluorescence levels three times greater than that of untransfected cells) contained significant amount of MTs (p<0.001) (Figure 4, B and C).

### TgICMAP1 binds MTs *in vitro*

**Figure 5:**
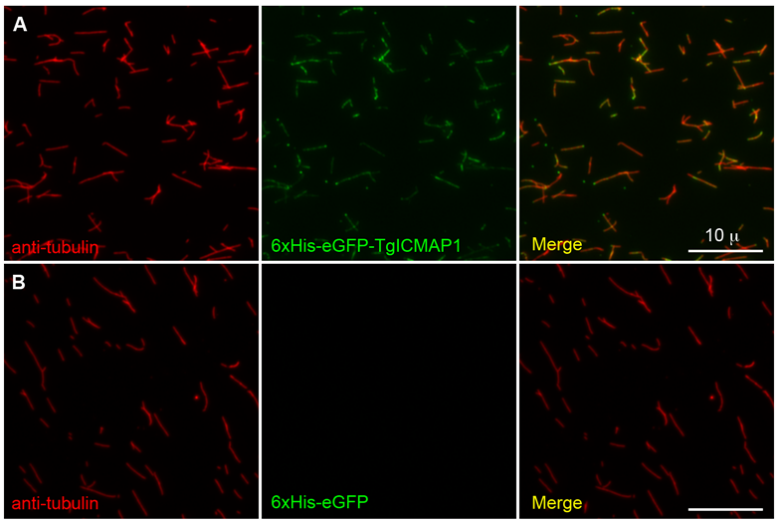
TgICMAP1 binds MTs *in vitro*. Recombinant 6xHis-eGFP-TgICMAP1 (*green*) (A) or 6xHis-eGFP (B) (*green*) were incubated with *in vitro* polymerized MTs, spun onto coverslips, and stained with mouse-anti-tubulin B-5-1-2 (*red*). 6xHis GFP-TgICMAP1 protein bound along the MT lattice. No MT binding was observed in 6xHis-eGFP control samples. Scale bars = 10 µm.

The fact that a *T. gondii* protein can bind to MTs in a “foreign” environment, the cytosol of mammalian cells, suggests that TgICMAP1 likely binds to the MTs directly rather than through other MT binding proteins. To determine if the interaction between TgICMAP1 and MTs is indeed direct, recombinant 6xHis-eGFP-TgICMAP1 was expressed and purified from *E. coli*, and incubated with *in vitro* polymerized MTs stabilized with the GTP analog GMPcPP. The 6xHis-eGFP-TgICMAP1 and MT suspension was then spun onto coverslips and MTs were visualized by staining with anti-tubulin antibody (Figure 5A). TgICMAP1 showed binding along the MT lattice. No MT binding was observed in the negative control sample, where 6xHis-eGFP [30] was used in place of 6xHis-eGFP-TgICMAP1 (Figure 5B).

### TgICMAP1 preferentially localizes to the intra-conoid MTs in *T. gondii*

**Figure 6:**
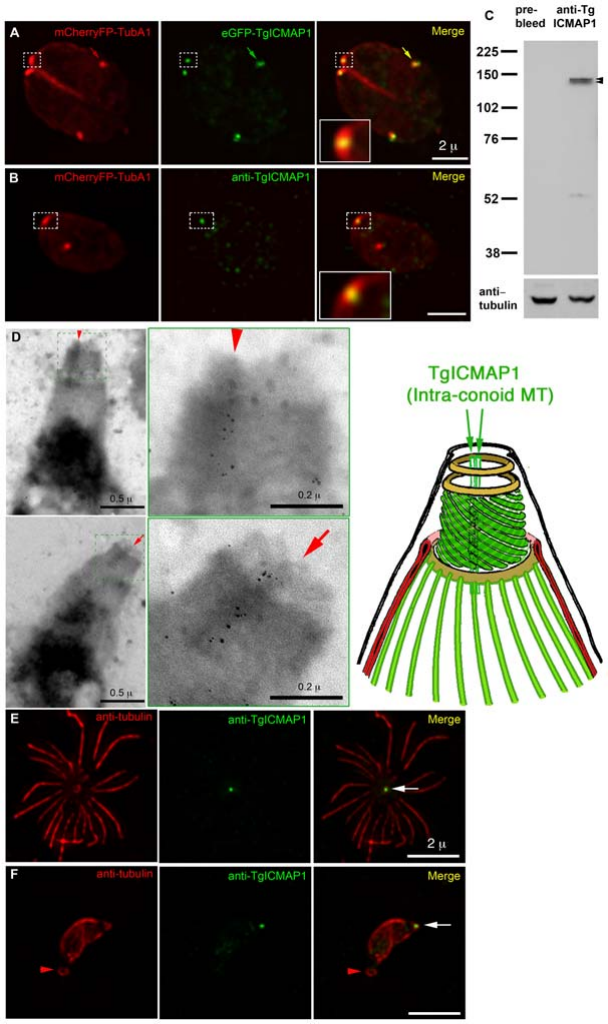
TgICMAP1 localizes to the intra-conoid MTs in *T. gondii*. (A) In *T. gondii*, eGFP-TgICMAP1 (*green*) localized to the apical complex of the parasite (*inset*), in a region narrower than the width of the conoid marked by mCherryFP-TubA1 (*red*). eGFP-TgICMAP1 also labeled the centriole/spindle pole area, which also incorporated mCherryFP-TubA1 (*arrows*). Inset is at 3× magnification. Scale bar = 2 µm. (B) Images of a parasite expressing mCherryFP-TubA1 (*red*) fixed and permeablized with methanol and labeled with anti-TgICMAP1 (*green*) show the prominent apical complex localization of endogenous TgICMAP1, which is similar to that of eGFP-TgICMAP1, but a specific localization in the centriole/spindle pole area could not be confirmed. The punctate labeling in the parasite body is likely to be the combined result of labeling for TgICMAP1 in the cytoplasmic pool and non-specific labeling, as some spots are seen outside the parasite. Inset is at 3× magnification. Scale bar = 2 µm. (C) Western blot of *T. gondii* total cell lysate showing that anti-TgICMAP1 antibody recognizes a ∼135 kDa protein doublet (*arrowheads*) consistent with the predicted size of TgICMAP1. The blot was stripped and reprobed with mouse-anti-tubulin B-5-1-2 to use α-tubulin as a loading control. (D) *Left*: Two EM images of Ca^2+^ ionophore–treated, deoxycholate-extracted RH parasites, which were immunogold-labeled with anti-TgICMAP1 antibody and negatively stained with phosphotungstic acid. Scale bars = 0.5 µm. *Middle*: Higher magnification of the apical complex regions of the parasites shown in left panels, showing that the intra-conoid MTs (*arrows* and *arrowheads*) are decorated with gold particles, recapitulating the distribution of fluorescence in Figure 6 A and B. Scale bars = 0.2 µm. *Right*: schematic diagram of the *T. gondii* apical complex. (E and F) Parasites were extracted with deoxycholic acid, and subsequently fixed and stained with anti-tubulin B-5-1-2 (*red*) and anti-TgICMAP1 (*green*). The cortical cytoskeleton of the parasite in E was mostly destroyed with the cortical MTs splaying around the apical complex (*white arrow*). In (F) the cortical cytoskeleton of the parasite was more intact and a ring-like structure stained with anti-tubulin antibody can be observed at the basal end of the parasite (*red arrowheads*). Scale bars = 2 µm.

Despite its ability to bind MT directly, TgICMAP1 only binds to a subset of tubulin containing structures in *T. gondii*. Colocalization studies showed that eGFP-TgICMAP1 predominately localized to a thin focal point at the apical end of the parasite (Figure 6A), much narrower in width when compared to that of the conoid, marked by co-expression of mCherryFP-TubA1 in the same cell. eGFP-TgICMAP1 also localized to the centriole/spindle pole area (arrows) (also marked by the expression of mCherryFP-TubA1 [8]), although the intensity of this labeling varied depending on the expression level of eGFP-TgICMAP1. To investigate the localization of endogenous TgICMAP1, an anti-TgICMAP1 antibody was produced (Figure 6B–F). Immunofluorescence assays using the anti-TgICMAP1 antibody confirmed that endogenous TgICMAP1 had similar apical localization as eGFP-TgICMAP1 in the apical complex, but a specific localization in the centriole/spindle pole area could not be confirmed (Figure 6B). By Western blot, the anti-TgICMAP1 antibody recognized a closely spaced doublet band around 135 kDa, the predicted size of TgICMAP1 (Figure 6C). We do not know why TgICMAP1 ran as a doublet in reducing SDS-PAGE, but post-translational modification is a potential cause, and we are currently investigating this possibility.

Because the conoid and the intra-conoid MTs are the only tubulin containing structures in the apical complex (Figure 1) [3], the highly restricted localization of TgICMAP1 in the apical complex strongly suggests that TgICMAP1 binds to the intra-conoid MTs. This was confirmed by electron microscopy, where the intra-conoid MTs were decorated with gold particles indicating the binding of anti-TgICMAP1 antibody (Figure 6D).

Consistent with our previous experience in immunolabeling apical complex proteins, where antigen accessibility was often an issue [7], harsh extraction conditions were needed for the successful labeling of the intra-conoid MTs with the anti-TgICMAP1 antibody. For extracellular parasites we found that 10 mM deoxycholate extraction gave the best labeling of the intra-conoid MTs with the anti-TgICMAP1 antibody (Figure 6, E and F, arrows). This condition often resulted in the loss of the banana-like shape of the parasite, likely due to the release of the cortical MTs from other parasite membrane-cytoskeletal elements (Figure 6E). However in cases where the basal end of the parasite held together, we were surprised to find anti-tubulin staining in a ring-like structure at the basal end of the parasite (Figure 6F, arrowhead). This is the first time that a tubulin containing structure has been observed at the basal end of the parasite. Recently, another group has also made a similar observation independently (Personal communication, Louis Weiss, Albert Einstein College, NY).

### The first 474 amino acids of TgICMAP1 are important for intra-conoid MT association in the parasite

**Figure 7:**
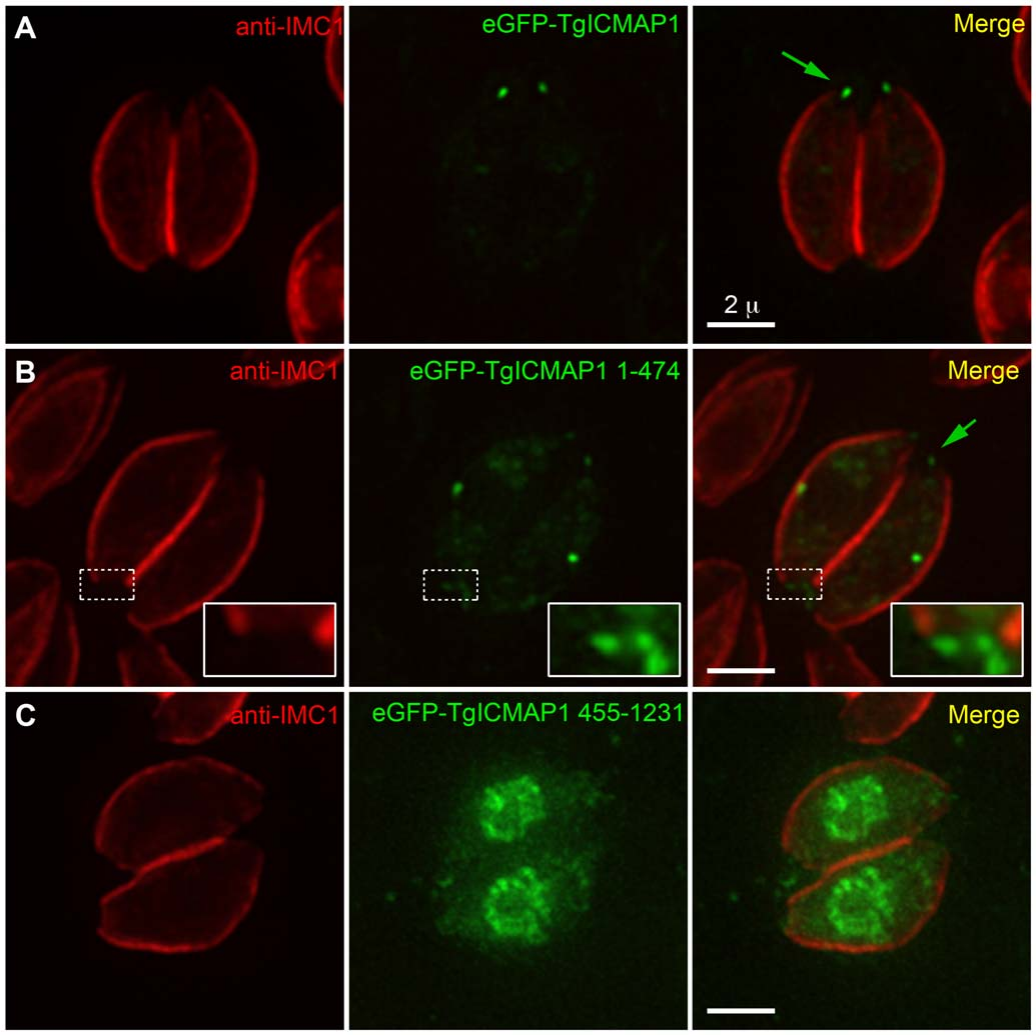
The first 474 amino acids of TgICMAP1 are important for intra-conoid MT association in *T. gondii*. Interphase parasites expressing eGFP fusions (*green*) of full-length TgICMAP1 (A), TgICMAP1^1–474^ (B), and TgICMAP1^455–1231^ (C) were labeled with anti-IMC1 antibody (*red*). EGFP-TgICMAP1^1–474^ is localized to the intra-conoid MTs (*green arrows*). However, it displays stronger localization to the basal complex (*inset*) in comparison to eGFP-TgICMAP1. Its cytoplasmic pool is also more prominent than that of eGFP-TgICMAP1. eGFP -TgICMAP1^455–1231^ is localized predominantly to the nucleus and cytosol. The insets are at 2.5× magnification. Scale bars = 2 µm.

To determine if the SMC containing N-terminal domain is important for MT binding, we made eGFP fusions of two TgICMAP1 truncation mutants and expressed them in *T. gondii*: eGFP-TgICMAP1^1–474^, a N-terminal truncation that contains the SMC-like coiled-coil domain, and eGFP-TgICMAP1^455–1231^, a C-terminal truncation that excludes most of the SMC-like domain (c.f. Figure 3B). Expression of eGFP-TgICMAP1^1–474^ recapitulated the intra-conoid MT localization of the full-length protein at the apical complex with, however, more prominent cytoplasmic pool localization (Figure 7A–B). Interestingly, eGFP-TgICMAP1^1–474^ also displayed basal complex labeling (Figure 7B, inset). In contrast, deletion of the SMC-like domain in eGFP-TgICMAP1^455–1231^ resulted in the complete loss of apical complex localization, with the truncated protein predominantly localizing to the nucleus (Figure 7C). Together, these data show that the first 474 amino acids of TgICMAP1 are essential for the intra-conoid MT association in *T. gondii*. The role of the C-terminus is less clear, but may be important for regulating the efficiency of TgICMAP1 targeting to the apical complex or may affect the binding affinity of TgICMAP1 for MTs.

### TgICMAP1 is a marker for monitoring the intra-conoid MT construction process during daughter formation

**Figure 8:**
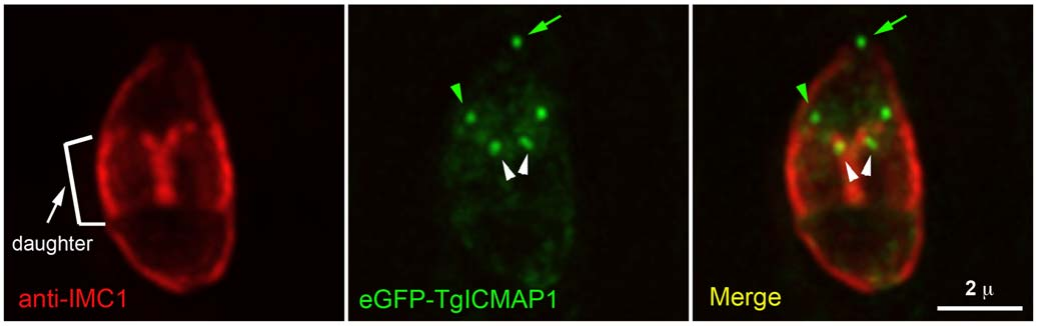
Daughter intra-conoid MTs are assembled during daughter formation within the mother cell. eGFP-TgICMAP1 expression (*green*) and anti-IMC1 staining (*red*) in a dividing parasite show that eGFP-TgICMAP1 is present at the intra-conoid MTs of both the mother (*green arrow*) and the daughter parasites (*green arrowhead*) as well as the duplicated centriole/spindle pole assembly (*white arrowheads*). Scale bar = 2 µm.

The apical complex is a highly intricate, motile cytoskeletal machine that is likely to be important in parasite invasion and replication [6]. Therefore, how the different parts of this machine are assembled together during daughter cell construction is of great interest. Due to the lack of EM evidence and appropriate markers, it is not known whether the two intra-conoid MTs are assembled into the apical complex when the daughters are still being constructed inside the mother or after the daughters have emerged from the mother and become independent entities. TgICMAP1, the first marker for the intra-conoid MTs, allowed us to address this question. Figure 8 shows the localization of eGFP-TgICMAP1 in a dividing parasite (*green*), where both the mother and two daughter cortical profiles were marked by an antibody recognizing IMC1 (*red*), a component of a protein meshwork underlying the parasite membrane pellicle [31]–[33]. In addition to the mother intra-conoid MTs, eGFP-TgICMAP1 was localized to the daughter intra-conoid MTs and the duplicated centriole/spindle pole assembly. Therefore, the daughter intra-conoid MTs are present in the daughter parasites as they are formed within the mother.

## Discussion

Unlike mammalian cells in culture where the majority of the MTs in the cell are highly dynamic, the tubulin containing structures in *T. gondii* are extraordinarily stable in interphase parasites. The apical complex and the cortical MTs, for example, remain intact after harsh detergent extractions (c.f. Figure 6D–F) [1], [3], [7] and are highly resistant to MT depolymerization drugs such as oryzalin and nocodazole [5]. The stability of these tubulin containing structures provides stiffness to the parasite cortex, thus they are important in maintaining the parasite shape and are likely to be important for basic parasite functions such as directional movement and invasion. MT binding proteins are central players in regulating MT stability and function. In this work, we report the identification of a new MT-binding protein in *T. gondii*, TgICMAP1, which is localized predominantly to the intra-conoid MTs − two highly stable MTs in the apical complex. When TgICMAP1 was ectopically expressed in mammalian cells, it significantly increased the resistance of MTs to nocodazole treatment, suggesting that TgICMAP1 might play a role in stabilizing the two intra-conoid MTs. We also found colocalization of TgICMAP1 with perinuclear MTs when TgICMAP1 was expressed at a high level in mammalian cells. It has been shown that perinculear MTs are more stable than other MTs, but it is unclear if their perinuclear localization is the cause or the consequence of their stability [34]–[36]. The colocalization of TgICMAP1 with this subset of MTs therefore could be due to the preference of TgICMAP1 in binding to these MTs (recognizing, for example, certain distinct biochemical or structural properties of these MTs) or an indirect result of the stabilization effect of TgICMAP1 on MTs. Right now we cannot distinguish between these two possibilities.

TgICMAP1 binds directly to the lattice of bare MTs polymerized *in vitro*, yet in *T. gondii* it preferentially binds to the intra-conoid MTs but not other tubulin containing structures, such as the conoid and the cortical MTs. How is this specific binding regulated? We showed previously that the conoid fibers are formed of protofilaments arranged in a “comma” like structure much different from that of a canonical MT [7] (c.f. Figure 1). This drastic structural difference between the conoid fibers and the intra-conoid MTs could well be the reason that TgICMAP1 is not found in the conoid. This cannot explain however, why TgICMAP1 does not bind to the cortical MTs, which are tubes with 13 protofilaments, just like the intra-conoid MTs. We speculate that the “masking” of the cortical MTs by other MT binding proteins might play a role in excluding TgICMAP1 from the cortical MTs. A second possibility is that differences in the post-translational modifications of tubulin in the intra-conoid MTs and the cortical MTs may affect the affinity of TgICMAP1 binding. In mammalian cells, for example, a number of MT-binding proteins and molecular motors preferentially bind detyrosinated MTs [37]–[41]. The identification of other proteins that are specifically localized to the intra-conoid MTs or the cortical MTs is necessary to test these hypotheses.

Protein secretion through the apical complex from the membrane-bound invasion organelles, rhoptries and micronemes, is essential for parasite invasion into the host cell [42]–[45]. MT based cytoskeleton mediates the majority of vesicle traffic in eukaryotic cells [46]–[49]. Based on electron microscopy evidence, it was proposed that the intra-conoid MTs might serve as tracks for the secretion of proteins from the apical complex during *T. gondii* invasion [3], [18]. Indeed, the length and position of the intra-conoid MTs are very consistent with their potential structural roles in secretion and the subsequent recycling of the lipid and proteins on the membrane of the emptied vesicles. However, due to the lack of knowledge of their protein composition other than tubulin, little is known about how the intra-conoid MTs function and how they are constructed during daughter formation. Using TgICMAP1 as a marker for the intra-conoid MTs, we showed that the intra-conoid MTs are formed during daughter parasite construction within the mother parasite. In addition, we noted that the anti-TgICMAP1 antibody recognized a doublet by Western blot, which may suggest that TgICMAP1 is post-translationally modified. Protein modifications, such as phosphorylation, have been shown to regulate the binding of MT-associated proteins to MT in vertebrates [50]. Therefore, it will be very interesting to elucidate whether TgICMAP1 is post-translationally modified, and understand whether such modification regulates TgICMAP1 function.

TgICMAP1 is the first molecular tool that can be used to dissect the biogenesis of the intra-conoid MTs. Future functional studies of TgICMAP1, such as the creation of TgICMAP1 deficient parasites and dominant negative mutants of TgICMAP1 will allow us to specifically interfere with the function of the intra-conoid MTs to understand the role of these two short MTs in the life of *T. gondii*.

## Materials and Methods

### Mammalian cell culture and transfection

HeLa cells were grown in DMEM (Cat#10569, Invitrogen, CA) containing 10% (v/v) Hyclone Bovine Calf Serum (BCS) (Cat#SH30087.03, Fisher Scientific, PA) and 1x antibiotic-antimycotic (Cat#15240, Invitrogen) at 37°C and 5% CO_2_. DNA (1 µg/well) was transfected into subconfluent HeLa cell monolayers grown in six well plates by FuGene6 transfection reagent (Cat #11815091001, Roche, IN) at a FuGene:DNA ratio of 3∶1 per manufacturer's instruction.

### Parasite culture and transfection

*T. gondii* RH tachyzoites were used in all experiments. The parasites were maintained by continuous passage in human foreskin fibroblasts (HFFs) as previously described [51]. For each *T. gondii* transfection, 30–40 µg of DNA was electroporated into 1×10^7^ extracellular parasites suspended in DMEM with 1% BCS. Electroporation was performed using a Harvard Apparatus BTX-BCM630 electroporator (Holliston, MA,) with the electroporation voltage set at 1480 V, capacitance set at 50 µF, and resistance set at 25 Ω.

### Construction of TgICMAP1 expression plasmids

#### 1. *T. gondii* expression plasmids

For the construction of pmin-eGFP-TgICMAP1, the TgICMAP1 coding region was amplified from the RH cDNA library (obtained as previously described [6]) using S124 and A125 primers (Table S1) digested with BamHI and AflII, and subcloned into pmin-eGFP-TgDLC plasmid [6] replacing the TgDLC/BglII-AflII fragment. The BamHI and BglII ligation created a sequence that cannot be re-cut by either BglII or BamHI. To introduce a unique BamHI site between the eGFP and TgICMAP1 for subsequent subcloning, a BamHI site downstream of the TgICMAP1 coding sequence on pmin-eGFP-TgICMAP1 was first removed by replacing the fragment between AflII and NotI sites with a PCR product amplified from pmin-eGFP-TgICMAP1 using PCR mutagenesis by overlap extension with primers S555, A555, S556, and A556 (Table S1). This resulted in the plasmid pmin-eGFP-TgICMAP1-minus-BamHI. A BamHI site is then created between eGFP and TgICMAP1 by replacing the fragment between NheI and NcoI sites in pmin-eGFP-TgICMAP1-minus-BamHI with a PCR product amplified from pmin-eGFP-TgICMAP1-minus-BamHI using PCR mutagenesis by overlap extension with primers S574, A574, S575, and A575 (Table S1). This resulted in the plasmid pmin-eGFP-TgICMAP1-BamHI-linker. The construction of pmin-eGFP-TgICMAP1-minus-BamHI and pmin-eGFP-TgICMAP1-BamHI-linker was carried out by Biomeans Inc. (Sugar Land, TX). pmin-eGFP-TgICMAP1^1–474^ was constructed by replacing eGFP-TgDLC/NheI-AflII in pmin-eGFP-TgDLC with eGFP-TgICMAP1^1–474^/NheI-HindIII from pc22-eGFP-TgICMAP1^1–474^ (see below). AflII and HindIII sites were filled-in by Klenow fragment before the ligation. pmin-eGFP-TgICMAP1^455–1231^ was constructed by replacing TgICMAP1/BamHI-AflII in pmin-eGFP-TgICMAP1-BamHI-linker with TgICMAP1^455–1231^/BamHI-AflII from pQE30-His-FLAG-eGFP-TgICMAP1^455–1231^ (see below).

#### 2. Bacterial expression plasmids

To generate a bacterial expression plasmid that has compatible sites with *T. gondii* expression plasmids, AflII and NheI sites were introduced into pQE30-DIP13 (a kind gift from Dr. Wolfgang Mages, Universität Regensburg, Germany) in two steps. First, an AflII site was introduced downstream of DIP13 by replacing the fragment between HindIII and PvuII in PQE30-DIP13 with a PCR product amplified from pQE30-DIP13 using PCR mutagenesis by overlap extension with primers S552, A552, S553, A553, S554, and A554 (Table S1). This resulted in the plasmid pQE30-DIP13_AflII. Second, a NheI site was introduced upstream of DIP13 by replacing the fragment between EcoRI and HindIII in pQE30-DIP13_AflII with a PCR product amplified from pQE30-DIP13_AflII using PCR mutagenesis by overlap extension with primers S552, A552, S576, and A576 (Table S1). This resulted in the plasmid pQE30-DIP13_AflII-NheI. The construction of pQE30-DIP13-AflII and pQE30-DIP13_AflII-NheI was carried out by Biomeans Inc. pQE30-6xHis-eGFP-TgICMAP1^1–474^ was constructed by replacing DIP13/NheI-HindIII in pQE30-DIP13_AflII-NheI with eGFP-TgICMAP1^1–474^/NheI-HindIII from pmin-eGFP-TgICMAP1-BamHI-linker. pQE30-6xHis-eGFP-TgICMAP1 was constructed by replacing DIP13/NheI-AflII in pQE30-DIP13_AflII-NheI with eGFP-TgICMAP1/NheI-AflII from pmin-eGFP-TgICMAP1-BamHI-linker. A FLAG tag was then introduced by amplifying a portion of the pQE30-6xHis-eGFP-TgICMAP1^1–474^ plasmid with primers pQE30 Forward and His-FLAG-NheI-AS (Table S1). The PCR product was digested with EcoRI and NheI and ligated into pQE30-His-eGFP-TgICMAP1^1–474^_EcoRI-NheI to form pQE30-6xHis-FLAG-eGFP-TgICMAP1^1–474^. To construct pQE30-His-FLAG-eGFP-TgICMAP1^455–1231^, TgICMAP1^455–1231^ was first amplified by PCR using pQE30-6xHis-eGFP-TgICMAP1 as a template and primers BamHI-TgICMAP1C-S and AflII-TgICMAP1C-AS (Table S1). The PCR product and pQE30-His-FLAG-eGFP-TgICMAP1^1–474^ were digested with BamHI and AflII restriction enzymes and ligated such that TgICMAP1^1–474^ was replaced with TgICMAP1^455–1231^. pBAD24-6xHis-TgICMAP1^1–474^ was constructed by ligating the 6xHis-TgICMAP1^1–474^ EcoRI-HindIII region from pQE30-6xHis-TgICMAP1^1–474^ into the EcoRI and HindIII sites of pBAD24 (A kind gift from Dr. Pat Foster, Indiana University, Bloomington).

#### 3. Mammalian cell expression plasmids

pc22-eGFP-TgICMAP1 was constructed by replacing the eGFP-TubA1 NheI-AflII fragment in pc22-eGFP-TubA1 (a kind gift from Dr. John Murray, University of Pennsylvania) with the eGFP-TgICMAP1 NheI-AflII fragment from pmin-eGFP-TgICMAP1 (see above). To construct pc22-eGFP-TgICMAP1^1–474^, pc22-eGFP-TgICMAP1 was digested with HindIII and AflII, filled in by Klenow fragment, and religated.

### Protein expression and purification

#### 1. Expression and purification of 6xHis-eGFP-TgICMAP1

Single BL21(DE3)pLysS bacterial colonies containing pQE30-6xHis-eGFP-TgICMAP1 plasmid were grown overnight in 50 ml LB containing 100 µg/ml of ampicillin, 50 µg/ml chloramphenicol (LB-amp-cap) at 37°C. Cultures were diluted 1∶25 into 750 ml LB-amp-cap until the OD_600_ reached ∼0.6. Cultures were induced with 1 mM isopropyl β-D-1-thiogalactopyranoside (IPTG) and grown overnight (∼18 hrs) at 16°C. Cells were then pelleted at 15,000 rpm for 5 minutes, resuspended in 40 ml of cold lysis buffer (8 mM Tris-Ac pH 7.5, 3 mM Trisbase, 100 mM KAc, 1 mM MgAc) containing 1% TX-100, 1.2 g of cell lytic express (∼1 vial, Cat#C1990, Sigma), 1 µM TAME (Cat#T4626: Sigma) and 1 µM PMSF (Cat#P7626:Sigma), and incubated at 4°C for 15 minutes. Cells were sonicated five times for 30 seconds each with 1 minute cooling between each cycle, then centrifuged at 15,000 rpm for 10 minutes at 4°C. 0.5 ml of Talon resin (Cat#635501, Clontech) equilibrated with lysis buffer was then added to the supernatant and gently mixed at 4°C for 2 hours. The resin was loaded on a gravity flow column (Cat#635513: Clontech) and then washed with 30 ml of wash buffer (lysis buffer containing 10 mM imidazole). Proteins were eluted with 250 µl of elution buffer (50 mM Tris-Cl pH 7.5, 250 mM imidazole, 300 mM KCl). Proteins were stored on ice and used within one week of purification.

#### 2. Expression and purification of recombinant 6xHis-TgICMAP1^1–474^ for antibody production

Single BL21(DE3)pLysS bacterial colonies containing pBAD24-6xHis-TgICMAP1^1–474^ plasmid were grown and lysed as described above with the following exceptions. Cultures were grown in LB-amp-cap containing 0.2% glucose and protein expression was induced with glucose-free LB-amp-cap containing 0.2% arabinose for four hours at 37°C [52]. After lysis and centrifugation the supernatant was discarded and insoluble proteins were solubilized using 6 M guanidine hydrochloride, 50 mM Hepes (pH 7) and 25 mM DTT (6 M guanidine HCl buffer) at 4°C for 1 hour. Insoluble materials were removed by centrifugation at 15,000 rpm for 10 minutes at 4°C. 1 ml of Talon resin equilibrated with 6 M guanidine HCl buffer was then added to the supernatant and gently mixed at 4°C for 2 hours. The resin was loaded on a gravity flow column (Cat#635606, Clontech) and then washed with successively lower amounts of guanidine hydrochloride (5 M, 4 M, 3 M, 2 M, 1 M 0.5 M and 0.25 M containing 50 mM Hepes and 25 mM DTT, pH 7). Proteins were eluted from the beads with 1 ml of elution buffer and the first and second eluates were pooled together and used to inject rats for the antibody production (Cocalico Biologicals Inc, Reamstown, PA).

### MT binding assays

Recycled bovine tubulin, guanylyl-(a,b)-methylene-diphosphonate (GMPcPP) (Jena Scientific, Jena, Germany), and 6xHis-eGFP were kind gifts from the lab of Claire Walczak (Indiana University, Bloomington). 10 µM clarified recycled tubulin was incubated with 0.5 mM final concentration of GMPcPP in BRB80 (80 mM piperazine-N,N-bix[2-ethanesulfonic acid] (PIPES) pH 6.8, 1 mM MgCl_2_, 1 mM EGTA, 1 mM DTT) at 37°C for 30 minutes [53], [54]. MTs were then pelleted at 45,000 rpm for 5 minutes in a Beckman TLA100 rotor at 30°C and resuspended in BRB80. MTs were diluted to 2 µM in BRB80 before use. Visual MT binding assays were performed essentially as described [53]. Reactions consisted of mixing 2 µM MTs and 1.2 µM 6xHis-eGFP-TgICMAP1 or 6xHis-eGFP together in a 1∶1 ratio and incubating for 15 minutes at 23°C. The reactions were fixed in 1% gluteraldehyde for 2 minutes, diluted with BRB80, and spun onto poly-L-lysine coated coverslips through a 10% (v/v) glycerol/BRB80 cushion at 14,000 rpm in a Beckman JS13.1 rotor for 45–90 minutes at 20°C. Coverslips were then dehydrated with −20°C methanol for five minutes and rehydrated with PBS (140 mM NaCl, 30 mM KCl, 2 mM K-Phosphate, 10 mM Na-Phosphate) with 0.1% Triton X-100 (TX-100) for 5 minutes. Finally, slides were blocked with PBS containing 2% BSA for 15 minutes, incubated consecutively with 1∶1000 dilution of mouse anti-tubulin B-5-1-2 and 1∶1000 dilution of goat anti-mouse Alexa 568 for 30 minutes each.

### Immunofluorescence

HeLa or mouse embryonic fibroblast cells were first fixed and permeabilized for 2 minutes at 37°C in PHEM buffer (60 mM PIPES, 28 mM Hepes, 10 mM EGTA, 8 mM MgSO_4_, pH 7.0) containing 3.7% (v/v) formaldehyde, 0.1% (v/v) gluteraldehyde, 1% (v/v) TX-100, fixed again in PHEM buffer containing 3.7% formaldehyde and 0.1% gluteraldehyde for 15 minutes, and then permeabilized again in PHEM buffer containing 1% TX-100 for 30 minutes. Cells were washed in PBS containing 1 mM MgCl_2_ (MgPBS) and quenched three times in PBS containing ∼10 mg sodium borohydride dissolved in PBS for two minutes each. Cells were then blocked in MgPBST (MgPBS, 0.02% (v/v) Tween-20) containing 2% (v/v) BSA for five minutes, incubated in primary antibody for 1 hour, washed three times in MgPBST and subsequently incubated in either goat anti-mouse Alexa 594 (Cat#: A11032, Molecular Probes, Invitrogen) or goat-anti-rat Alexa 647 (Cat#: A21247, Molecular Probes, Invitrogen) diluted 1∶1000 in MgPBS with 2% BSA, or incubated in donkey-anti-rat X-rhodamine (Cat#: 712-295-153, Jackson ImmunoResearch Laboratories, Inc, West Grove, PA) diluted 1∶500 in MgPBS with 1% BSA for 60 minutes. Primary antibody dilutions were as follows: mouse anti-tubulin B-5-1-2, 1∶1000 (Cat#T6074, Sigma); mouse anti-acetylated tubulin, 1∶400 (Cat#MMS-413R, Covance); rat anti-tubulin YL1/2 hybridoma supernatant, 1∶1 (A kind gift from Dr. John Murray, University of Pennsylvania); rat anti-tubulin YL1/2 ascites, 1∶800 (Cat#: MCA77G, AbD Serotec, Raleigh, NC). Coverslips were mounted on slides using ProLong Gold Antifade Reagent (Cat#P36930, Molecular Probes, Invitrogen). All incubations were carried out at room temperature unless otherwise stated. For methanol fixation, cells were washed three times at 37°C in PBS, fixed and permealized for 5 minutes in −20°C methanol. Cells were then washed three times in MgPBS. The rest of the procedure for immunofluorescence was the same as described above.

For immunolabeling of intracellular parasites, *T. gondii* growing in HFF monolayer were fixed in either 3.7% formaldehyde in PBS for 15 minutes and permeabilized with 0.25% TX-100 in PBS for 15 minutes at room temperature or −20°C methanol for 5 minutes. Cells were blocked with 2% BSA in PBS for 5 minutes at room temperature and then incubated in primary and subsequently secondary antibody solutions for 60 minutes each. Primary antibody dilutions were as follows: mouse anti-IMC1, 1∶1000 (A kind gift from Dr. Gary Ward, University of Vermont); mouse anti-tubulin B-5-1-2, 1∶1000; rat anti-TgICMAP1, 1∶100 (produced by Cocalico Biological, Inc.). Goat anti-mouse Alexa 568 (Cat#: A11031, Molecular Probes-Invitrogen) and goat anti-rat Alexa 488 (Cat#: A11006, Molecular Probes-Invitrogen) secondary antibodies were used at 1∶1000 dilution.

For immunolabeling of extracellular parasites, 1×10^7^ parasites were first suspended in PBS containing 5 µM calcium ionophore, A23187 (Cat#C7522, Sigma), and then spotted onto clean parafilm and overlayed with poly-lysine coated coverslips for 30 minutes. The purpose for having A23187 in the suspension was to induce conoid extension, which is calcium dependent. Parasites were then extracted in 10 mM deoxycholate in PBS for 25 minutes, and fixed in 3.7% formaldehyde in PBS for 15 minutes. Coverslips were blocked with 2% BSA in PBS for five minutes. The cells were then incubated for 60 minutes in mouse anti-tubulin B-5-1-2 diluted 1∶1000 and rat anti-TgICMAP1 diluted 1∶100 and subsequently in goat anti-rat Alexa 488 and goat anti-mouse Alexa 568 diluted 1∶1000.

### MT stability assay in HeLa cells

Untransfected and pc22-eGFP-TgICMAP1 transfected HeLa cells were treated with 2 µM nocodazole (Cat#M1404, Sigma) in DMEM with 10% BCS for 4 hours at 37°C and 5% CO_2_. Slides were then fixed and processed as described above in the *Immunofluorescence* section. 50–100 untransfected control or transfected cells were scored from the same coverslip as either containing high or low levels of MTs (The levels of MTs were determined qualitatively. Representative images are shown in Figure 4C.), and the average percentage of cells containing high levels of MTs was determined from three independent experiments. Quantification of TgICMAP1 expression level was performed using SoftWoRx (Applied Precision, Inc). Cells transfected with pc22-eGFP-TgICMAP1 were assigned to high or low expression groups using three times the average fluorescence of untransfected cells as the threshold. Statistical analysis (Student's t-test) was performed using GraphPad Prism version 5.0 and a difference was considered to be significant when p<0.01.

### Light microscopy

3D image stacks were collected at room temperature at z-increments of 0.3 µm using an Applied Precision Delta Vision imaging station constructed on an Olympus IX-70 inverted microscope base. A 100× oil immersion lens (1.4 NA) and immersion oil at refractive index 1.518 were used for all the imaging. Deconvolved images were computed using the point-spread functions and software supplied by the manufacturer. Fluorescent images are maximum intensity projections of deconvolved 3D stacks. The brightness and contrast of images used in the final figures were optimized for color prints.

### ImmunoGold Labeling

3×10^7^ RH parasites were first suspended in 5 µl PBS containing 5 µM calcium ionophore, A23187, and then spotted onto clean parafilm and overlayed with a carbon-film–coated nickel grid for 1 hour in a humid chamber. Parasites were then permeabilized with 10 mM DOC in PBS for 25 minutes and fixed with 3.7% formaldehyde in PBS, and washed 3×5 minutes with PBS, followed by 10 minutes of blocking in 5% BSA +0.1% (v/v) fish gelatin. Free aldehyde groups were blocked by incubation with 50 mM glycine in PBS for 15 minutes (pH 7.5) followed by 15 minute incubation with 0.1% (w/v) NaBH_4_ in PBS. Grids were washed twice with PBS, blocked again with 5% BSA +0.1% fish gelatin in PBS for 30 minutes, washed 2×5 minutes with incubation buffer (0.8% BSA, 0.1% fish gelatin in PBS plus 10 mM NaN_3_), incubated for ∼2 hours with primary antibody (rat anti-TgICMAP1), diluted 1∶100 in incubation buffer; washed 6×5 minutes in incubation buffer, inverted on 15 µl drops of secondary antibody (goat anti–rat IgG-conjugated with ultra-small gold (EMS Inc Cat#25181)), diluted 1∶50 in incubation buffer; incubated ∼19 hours at 4°C, then washed with incubation buffer as follows: 3×1 minute, 2×10 minutes, 4×5 minutes, and then 5×1 minute with PBS. The samples were post-fixed for 5 minutes with 1% glutaraldehyde in PBS and washed in distilled water 3×5 minutes. Silver enhancement was carried out using the HQ silver enhancement kit (Nanoprobes) by floating grids on mixtures of the initiator, activator, and modulator for 6 minutes in a light-tight chamber, then washing briefly with distilled water once followed by 2×5 minutes washes in distilled water. Grids were negatively stained using 2% phosphotungstic acid (pH 7.0).

### Western blot

5×10^6^ extracellular parasites were lysed by incubating in 1x sample buffer (62.5 mM Tris pH 6.8, 2% (w/v) sodium deodeoyl sulfate, 10% (v/v) glycerol, ∼0.5 mg of bromophenol blue) containing 50 mM DTT at 100°C for 10 minutes. Proteins were resolved on 4–12% gradient bis-tris gels (Cat#NP0332, Invitrogen) at 200 V for 1 hour. Proteins were transferred to polyvinylidene fluoride (PVDF) membrane using the XCell^TM^ blot module (Cat#EI9051, Invitrogen) per manufacturers instructions. Western blot was carried out using BM chemiluminescence Western blotting kit (Cat#1152709001, Roche) per the instructions of the manufacturer. Washes were carried out in TBS (20 mM Tris base pH 7.4, 150 mM NaCl) containing 0.1% (v/v) Tween-20 (Sigma Cat#P7949). Rat anti-TgICMAP1 was diluted 1∶250 and mouse-anti-tubulin B-5-1-2 was diluted 1∶2,000 in TBS containing 0.5% (v/v) blocking buffer (Cat#1152709001, Roche). Goat anti-rat IgG HRP (Cat#NA935V, GE Healthcare, United Kingdom) was diluted 1∶1000 and goat anti-mouse/rabbit HRP (Cat#1152709001, Roche) was diluted 1∶20,000 in TBS containing 0.5% blocking buffer.

## Supporting Information

Table S1 Primer sequences used for the construction of plasmids used in this paper. Abbreviations: S; sense primer, AS; anti-sense primer.
(0.05 MB DOC)

Figure S1 NcICMAP1 is homologous to TgICMAP1. ClustalW alignment of TgICMAP1 and NcICMAP1, which share 82% identity and 89% similarity. Sequence homologous to the coiled-coil SMC domain is highlighted in red. “*” indicates conserved amino acids; “:” indicates conserved substitution; “.” indicates semi-conserved substitution. EuPathDB accession numbers are TgICMAP1: TgME49_039300, NcICMAP1: Nc_LIV_ 070470.
(0.74 MB TIF)
