# Supplementary material for: TgICMAP1 Is a Novel Microtubule Binding Protein in Toxoplasma gondii
Source: PLoS One. 2009 Oct 12;4(10):e7406. doi: 10.1371/journal.pone.0007406 (PMC2758671; doi:10.1371/journal.pone.0007406)
Supplement: Table S1 — Primer sequences used for the construction of plasmids used in this paper. Abbreviations: S; sense primer, AS; anti-sense primer. (0.05 MB DOC) [file pone.0007406.s001.pdf]

Table S1.

Primer Name and Sequence:	Used for the construction of:
S124: ATGCGGATCCATGGCAGATCTGACACTCGAGCCCCG<br>A125: ATCGCTTAAGTTACCACCAGCCCCAACTGGAACTCTC	pmin-eGFP-TgICMAP1
S555: GCCGGCGAACGTGGCGAGA<br>A555: CGGATAAAGAGGCAGTCGAGGCG;<br>S556: GCCGCTCTAATCCCCCTCCACCGCGGTGT<br>A556: GAGGGGGATTAGAGCGGCCGCCACCGC	pmin-eGFP-TgICMAP1-minus-BamHI
S574: GCCATGGATCCGAGTCCGGACTTGTACAGCTC<br>A574: GACTCGGATCCATGGCAGATCTGACAC<br>S575: GGTACCGAGTCGTCATGTCTG<br>A575: CGCTTTAGTAGTGCGTTTCTGC	pmin-eGFP-TgICMAP1FL_BamHIlinker.
S552: TAGATTCAATTGTGAGCGGATAAC<br>A552: AGGCGGGCAAGAATGTG<br>S553: GAAAGCTTAAGTAGCTGAGCTTGGACTCCTGT<br>A553: GCTCAGCTACTTAAGCTTTCAAGAGCTGGCC<br>S554: GAGAATCCAAACTAGCTTGGCGAGATTTTCAG<br>A554:CCAAGCTAGTTTGGATTCTCACCAATAAAAAACG	pQE30-DIP13_AflII
S552: TAGATTCAATTGTGAGCGGATAAC<br>A552: AGGCGGGCAAGAATGTG<br>S576: TCACGCTAGCGGATCCATGTCTGCTCAAGGC<br>A576: GGATCCGCTAGCGTGATGGTGATGGTGATGCG	pQE30-DIP13_AflII-NheI
BamHI-TgICMAP1C-S:<br>AGCGGATCCACGCTGGAGGCAAAAGTGAGTGAGC<br>AflII-TgICMAP1C-AS:<br>ATCGCTTAAGTTACCACCAGCCCCAACTGGAACTCTC	pQE30-His-FLAG-eGFP-TgICMAP1$^{455-1231}$
pQE30 Forward: CCCGAAAAGTGCCACCTG<br>His-FLAG-NheI-AS:<br>ACGCTAGCCTTATCGTCGTCATCCTTGTAATCGTGATGGTGATGGTGATG	pQE30-His-FLAG-eGFP-TgICMAP1$^{1-474}$
