# Supplementary figures and images for: TgICMAP1 Is a Novel Microtubule Binding Protein in Toxoplasma gondii
Source: PLoS One. 2009 Oct 12;4(10):e7406. doi: 10.1371/journal.pone.0007406 (PMC2758671; doi:10.1371/journal.pone.0007406)

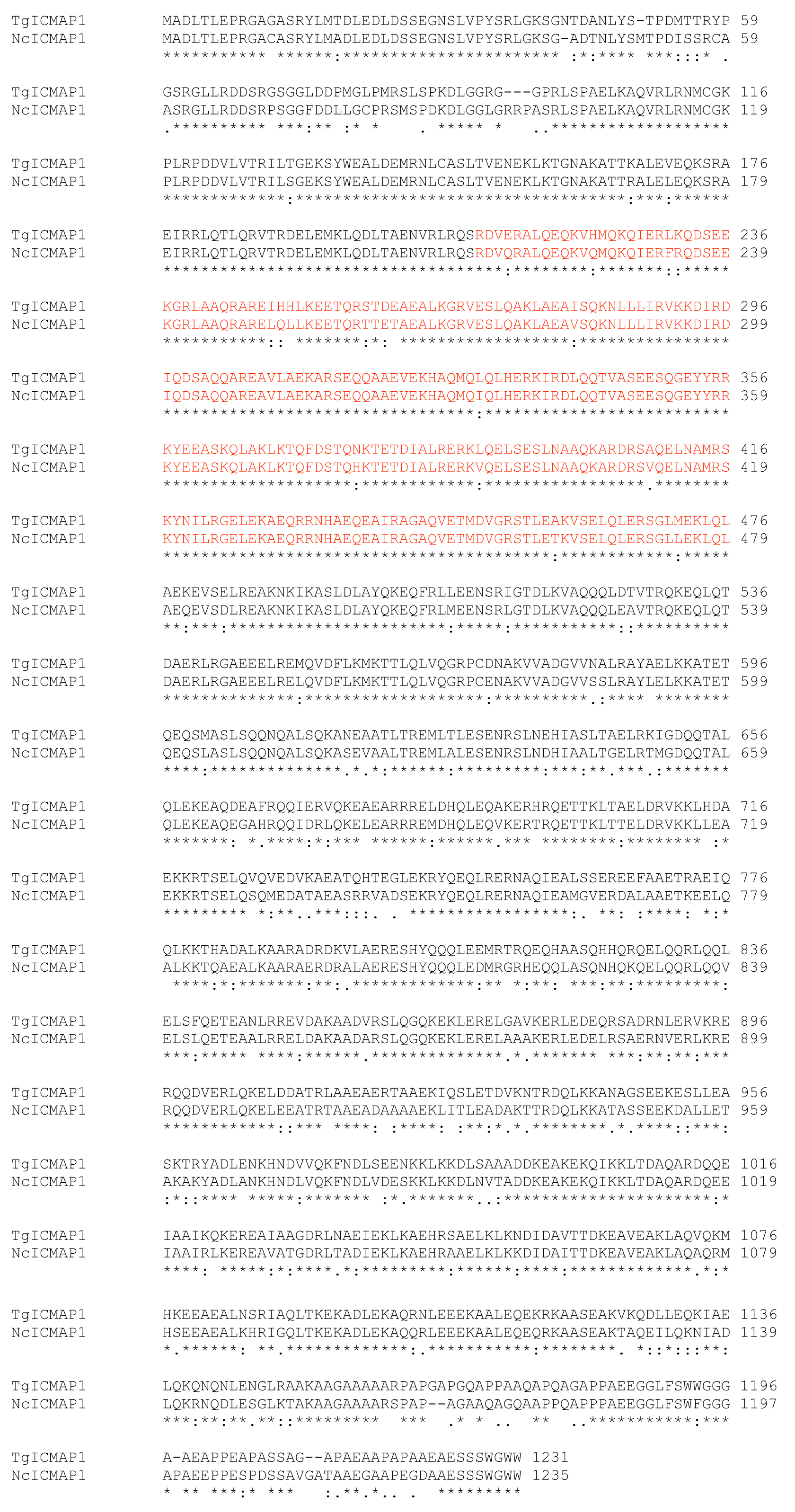

Supplement: Figure S1 — NcICMAP1 is homologous to TgICMAP1. ClustalW alignment of TgICMAP1 and NcICMAP1, which share 82% identity and 89% similarity. Sequence homologous to the coiled-coil SMC domain is highlighted in red. “*” indicates conserved amino acids; “:” indicates conserved substitution; “.” indicates semi-conserved substitution. EuPathDB accession numbers are TgICMAP1: TgME49_039300, NcICMAP1: Nc_LIV_ 070470. (0.74 MB TIF) [file pone.0007406.s002.tif]
